# Lack of awareness of erectile dysfunction in many men with risk factors for erectile dysfunction

**DOI:** 10.1186/1471-2490-10-18

**Published:** 2010-11-05

**Authors:** Ridwan Shabsigh, Joel Kaufman, Michelle Magee, Dana Creanga, David Russell, Meeta Budhwani

**Affiliations:** 1 Maimonides Medical Center, Brooklyn, NY, and Columbia University, New York, NY, USA; 2 Urology Research Options, Aurora, CO, USA; 3 MedStar Research Institute, Washington, DC, USA; 4 Consultant to Pfizer Inc, New York, NY, USA; 5 Pfizer Inc, New York, NY, USA

## Abstract

**Background:**

Men with erectile dysfunction often have concurrent medical conditions. Conversely, men with these conditions may also have underlying erectile dysfunction. The prevalence of unrecognized erectile dysfunction in men with comorbidities commonly associated with erectile dysfunction was determined in men invited to participate in a double-blind, randomized, placebo-controlled trial of sildenafil citrate.

**Methods:**

Men ≥30 years old presenting with ≥1 erectile dysfunction risk factor (controlled hypertension, hypercholesterolemia, smoking, metabolic syndrome, stable coronary artery disease, diabetes, depression, lower urinary tract symptoms, obesity [body mass index ≥30 kg/m^2^] or waist circumference ≥40 inches), and not previously diagnosed with erectile dysfunction were evaluated. The screening question, "Do you have erectile dysfunction?," with responses of "no," "yes," and "unsure," and the Erectile Function domain of the International Index of Erectile Function (IIEF-EF) were administered.

**Results:**

Of 1084 men screened, 1053 answered the screening question and also had IIEF-EF scores. IIEF-EF scores indicating erectile dysfunction occurred in 71% (744/1053), of whom 54% (399/744) had moderate or severe erectile dysfunction. Of 139 answering "yes," 526 answering "unsure," and 388 answering "no," 96%, 90%, and 36%, respectively, had some degree of erectile dysfunction. The mean±SD (range) number of risk factors was 2.9 ± 1.7 (3-8) in the "yes" group, 3.2 ± 1.7 (3-9) in the "unsure" group, and 2.6 ± 1.5 (2-8) in the "no" group.

**Conclusion:**

Although awareness of having erectile dysfunction was low, most men with risk factors had IIEF-EF scores indicating erectile dysfunction. Erectile dysfunction should be suspected and assessed in men with risk factors, regardless of their apparent level of awareness of erectile dysfunction.

**Trial registration:**

ClinicalTrials.gov Identifier NCT00343200.

## Background

Erectile dysfunction affects quality of life and may be associated with depression[1-4]. Men with erectile dysfunction often have other comorbidities such as diabetes, hypertension, and coronary artery disease[5-8]. Conversely, men consulting with their physician for comorbidities or other risk factors for erectile dysfunction may also have underlying erectile dysfunction, which may or may not be recognized.

Erectile dysfunction is defined as the inability to attain or maintain an erection sufficient for satisfactory sexual performance[9]. However, men who experience a change in their ability to achieve an erection might not immediately recognize that erectile dysfunction is the problem. The quality of men's erections deteriorates gradually over time. Consequently, men may be uncertain whether their erectile difficulties are permanent or temporary[10] and may wait to see if the erectile dysfunction resolves on its own[11]. Alternatively, the stigma or embarrassment of having erectile dysfunction symptoms may lead to denial of the problem[10,11].

We hypothesized that men with comorbidities and risk factors associated with erectile dysfunction frequently have this condition but might deny it and not identify themselves as erectile dysfunction sufferers. The current report discusses the design and outcome of a screening strategy for men with erectile dysfunction-associated comorbidities and risk factors who do not self-identify as having erectile dysfunction. The objective was to create a profile of these men by describing the general characteristics (demographics, comorbidities, and risk factors) of men who answered the question, "Do you have erectile dysfunction?" with "yes," "no," or "unsure" responses. Such information is needed in order to allow formulation of strategies to identify previously unrecognized or undiagnosed erectile dysfunction in order that it may be addressed as a medical condition.

## Methods

Men were recruited for a men's health study without mention of erectile dysfunction. At the screening visit for this sildenafil flexible-dose, double-blind, placebo-controlled trial,[12] written informed consent was obtained, and demographic data and the patient's history of risk factor(s) were collected. The protocol was approved by the Institutional Review Board of each participating center, and the study was conducted in compliance with the ethical principles originating in or derived from the Declaration of Helsinki and in compliance with all International Conference on Harmonization Good Clinical Practice Guidelines. Men ≥30 years of age who presented with at least 1 risk factor or comorbidity for erectile dysfunction (controlled hypertension, hypercholesterolemia, smoking, metabolic syndrome, stable coronary artery disease, diabetes, depression, lower urinary tract symptoms [LUTS], obesity [defined as body mass index [BMI] ≥30 kg/m^2^] or waist circumference ≥40 inches) and who had not been previously diagnosed with erectile dysfunction were eligible for screening.

Key exclusion criteria included hypotension, current or anticipated nitrate or nitric oxide donor treatment, significant cardiovascular disease within the past 3 months, and previous use of more than 6 doses of any phosphodiesterase type 5 inhibitor.

Men were asked the screening question, "Do you have erectile dysfunction?" and administered the Erectile Function domain of the International Index of Erectile Function (IIEF-EF)[13]. Those who answered "no" or "unsure" to the erectile dysfunction question and who had any degree of erectile dysfunction (scored ≤25 out of 30 on the IIEF-EF) [14] were eligible for inclusion into the double-blind, placebo-controlled trial[12]. The results of the screening analysis are reported here.

## Results

**Figure 1:**
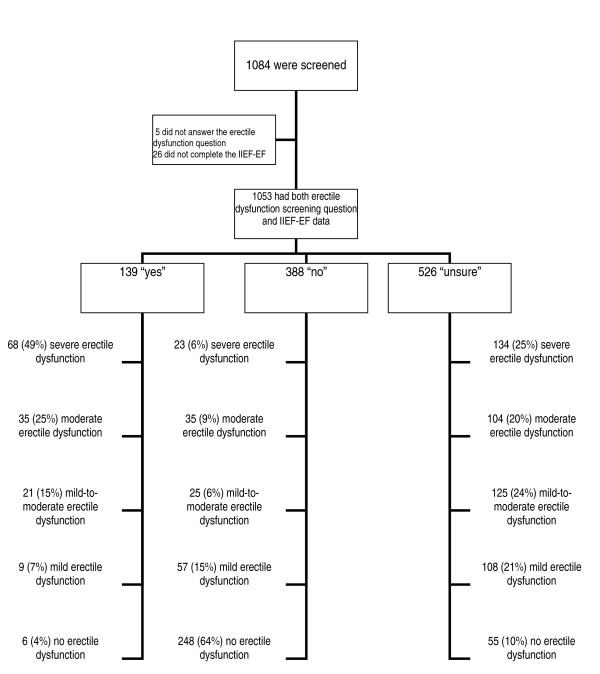
**Erectile dysfunction severity by screening question response**. IIEF-EF = Erectile Function domain of the International Index of Erectile Function. Using the IIEF-EF, erectile dysfunction severity categories are no ED (score ≥26 out of 30) mild (score 22-25), mild-to-moderate (score 17-21), moderate (score 11-16), and severe (score ≤10)[13,14].

Of the 1084 men screened, 1079 responded to the erectile dysfunction screening question and 1053 also had IIEF-EF scores (Figure 1). Overall, IIEF-EF indicative of erectile dysfunction were noted in 71% (744/1053) of men, of whom 54% (399/744) had moderate or severe erectile dysfunction (IIEF-EF score ≤16), 23% (171/744) had mild-to-moderate erectile dysfunction (IIEF-EF score 17-21), and 23% (174/744) had mild erectile dysfunction (IIEF-EF score 22-25).

One hundred thirty-nine men responded "yes" to the erectile dysfunction screening question and also completed the IIEF-EF; of these, 96% had IIEF-EF scores consistent with some degree of erectile dysfunction. Of those who answered "no" to the screening question (388/1053), 36% had also had IIEF-EF scores which indicated some degree of erectile dysfunction. Of those who answered "unsure" to the screening question (526/1053), 90% had IIEF-EF scores which indicated some degree of erectile dysfunction.

Although the mean age of the groups with "no" or "unsure" responses was 50 and 52 years, respectively, there were more men 45 years or older in the "unsure" group (75%) (Table 1). The "yes" group had the highest mean age (59 years), and the greatest percentage of those aged 65 years or older (32%).

**Table 1:** Patient Characteristics by Response to the Screening Question, "Do You Have Erectile Dysfunction?"

	Yes (n = 139)	No (n = 388)	Unsure (n = 526)	Total **(N = 1053)**^**†**^
Mean age, y (range)	59 (36-82)	50 (29-78)	52 (29-85)	52 (29-85)
Age distribution, n (%)				
18-44 y	9 (6)	127 (33)	129 (25)	265 (25)
45-64 y	86 (62)	222 (57)	343 (65)	651 (62)
≥65 y	44 (32)	39 (10)	54 (10)	137 (13)
Race, n (%)				
White	113 (81)	317 (82)	380 (72)	810 (77)
Black	12 (9)	48 (12)	95 (18)	155 (15)
Asian	3 (2)	3 (1)	3 (1)	9 (1)
Other	9 (6)	20 (5)	48 (9)	77 (7)
Missing	2 (1)	0	0	2 (< 1)
Mean weight, kg (range)*	92.0 (64.4-143.3)	95.9 (57.6-155.6)	99.7 (56.7-195)	97.4 (56.7-195)
Mean height, cm (range)*	177 (160-193)	178 (152-193)	178 (150-198)	178 (150-198)

*Data available for 971 subjects.

^†^Patients with both erectile dysfunction screening question and Erectile Function domain of the International Index of Erectile Function data.

**Figure 2:**
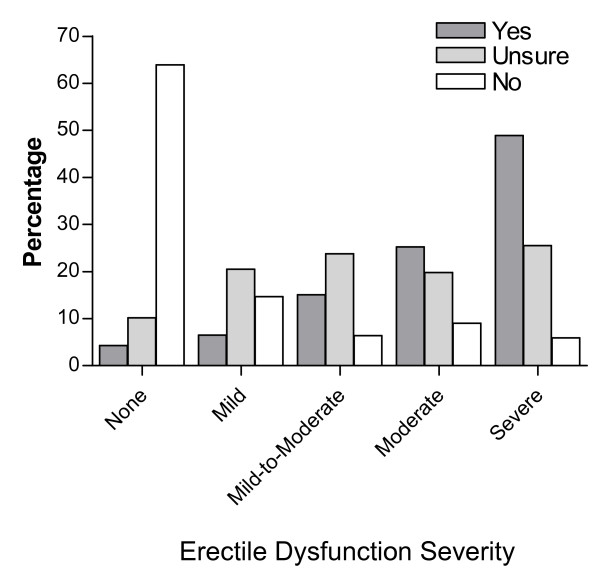
**Erectile dysfunction severity at screening**. Erectile dysfunction severity at screening was based on Erectile Function Domain of the International Index of Erectile Function score in men who responded "yes," "no," or "unsure" to the question, "Do you have erectile dysfunction?" Percentages with each erectile dysfunction severity category were calculated within the individual response groups ("yes," "no," or "unsure"). Erectile Function domain of the International Index of Erectile Function severity categories are no ED (score ≥26 out of 30), mild (score 22-25), mild-to-moderate (score 17-21), moderate (score 11-16), and severe (score ≤10)[13,14].

A correct erectile dysfunction diagnosis was more common for men who answered the screening question, "Do you have erectile dysfunction?" with the response of "yes" or "no" (Figure 2). Men who answered "yes" tended to have more severe erectile dysfunction, with 49% having severe erectile dysfunction (IIEF-EF score ≤10) and only 4% having no erectile dysfunction (IIEF-EF score ≥26). Men who answered "no" tended to have no or less severe erectile dysfunction, with only 6% having severe erectile dysfunction and 64% having no erectile dysfunction. The severity of erectile dysfunction in men who were unsure was almost equally distributed among the categories.

The mean number of erectile dysfunction risk factors was similar for all 3 erectile dysfunction screening response groups, with the mean ± SD of 2.9 ± 1.7 (range, 3-8) in the "yes" group, 2.6 ± 1.5 (range, 2-8) in the "no" group, and 3.2 ± 1.7 (range, 3-9) in the "unsure" group. Within each response group, hypertension, hypercholesterolemia, BMI ≥30 kg/m^2^, and waist circumference ≥40 inches occurred most frequently. For each risk factor, an "unsure" response was most common (48%-62%; vs "no," 23%-39%; vs "yes," 9%-27%) (Table 2).

**Table 2:** Erectile Dysfunction Screening Response Within Each Risk Factor Group*

		Yes, %	No, %	Unsure, %
Risk Factor	n	(n = 139)	(n = 388)	(n = 526)
Hypertension	552	15	33	52
Hypercholesterolemia	545	13	39	48
Obesity^†^	477	9	36	55
Smoking	400	9	32	60
Waist ≥40 inches	374	11	32	58
Diabetes	234	23	26	52
Depression	227	9	29	62
LUTS	119	19	26	56
Coronary artery disease	62	27	23	50
Metabolic syndrome	43	16	30	54

BMI = body mass index; LUTS = lower urinary tract symptoms.

*Patients with both erectile dysfunction screening question and Erectile Function domain of the International Index of Erectile Function data.

**^†^**BMI ≥30 kg/m^2^.

**Figure 3:**
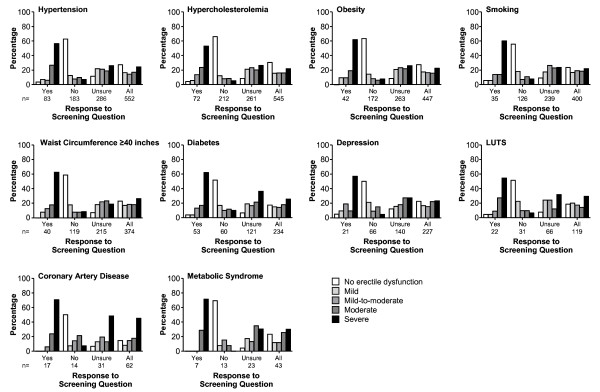
**Comparison of erectile dysfunction severity in comorbidity subgroups by screening question response**. LUTS = lower urinary tract symptoms. Erectile Function domain of the International Index of Erectile Function severity categories are no ED (score ≥26 out of 30), mild (score 22-25), mild-to-moderate (score 17-21), moderate (score 11-16), and severe (score ≤10)[13,14].

The erectile dysfunction severity profiles for each response group for the individual comorbidities generally reflected the pattern observed in the overall population; most men who answered "no" to the screening question had no erectile dysfunction or mild erectile dysfunction, those who answered "yes" had mostly moderate and severe erectile dysfunction, and those who answered "unsure" had erectile dysfunction severity that was almost equally distributed across the erectile dysfunction severity categories (Figure 3). However, in the subgroups of men with coronary artery disease and those with diabetes, a higher proportion of men in the "unsure" groups had severe erectile dysfunction compared with the other comorbidities.

## Discussion

Our screening for erectile dysfunction among men with an erectile dysfunction-associated comorbidity and risk factors showed a high prevalence of erectile dysfunction diagnosed with the IIEF-EF (71%), with more than half of these (54%) having moderate or severe dysfunction, 23% having mild-to-moderate dysfunction, and 23% having mild dysfunction. This supports recently reported results showing that even mild erectile dysfunction is an important indicator of risk for underlying disease associated with erectile dysfunction, results from the first prospective, randomized, double-blind trial to assess treatment with a phosphodiesterase type 5 inhibitor in a population of sexually dissatisfied men selected for mild ED diagnosed with the IIEF-EF[15]. That landmark trial also showed that sildenafil treatment was efficacious and well tolerated and the men were highly satisfied with their treatment[16]--thus validating both early intervention and the IIEF-EF diagnostic classification of mild erectile dysfunction in men who (per the inclusion criteria) have normal gonadal function, have regular sexual activity, and are sexually dissatisfied. Sexual dissatisfaction or "bother", which can be assessed using the Erection Distress Scale[17] or the Overall Satisfaction domain of the IIEF, may be an important part of the equation in a man's self-perception of erectile dysfunction, regardless of the severity of the dysfunction.

In the current trial, the distribution of erectile dysfunction severity differed by answer to the erectile dysfunction screening question, with a majority of men who answered "yes" having mostly moderate to severe erectile dysfunction. Likewise, men who answered "no" to the screening question had mostly no or mild erectile dysfunction (IIEF-EF score ≥22), although 6% of men who answered "no" had severe dysfunction according to the IIEF-EF assessment (IIEF-EF score ≤10). Men who were unsure whether they had erectile dysfunction most often did have some degree of erectile dysfunction, but the severity was variable.

Interestingly, a higher percentage of men in the "unsure" and overall groups with coronary artery disease or diabetes had severe erectile dysfunction compared with the other erectile dysfunction severity categories. The results for coronary artery disease are similar to those from a previous study showing 14% mild, 21% mild-to-moderate, 14% moderate, and 51% severe erectile dysfunction in men admitted to the emergency room with acute coronary syndrome and subsequently diagnosed with coronary artery disease[8]. Likewise, a study in diabetic men with erectile dysfunction showed that these men had significantly lower scores on the IIEF-EF than men without diabetes, with a mean IIEF-EF score of 6 (severe erectile dysfunction)[18].

A limitation of the trial design was that the reasons for men's specific answers to the screening question were not further investigated. However, studies investigating treatment-seeking behavior of men with erectile dysfunction and those assessing men's sexual attitudes and beliefs may serve to provide insight into how men react when they begin to experience erectile dysfunction as well as why some men may not recognize that they have erectile dysfunction.

Erectile dysfunction may produce a profound sense of loss[10]. Men may try to make sense of the cause of their erectile dysfunction, which may include guilt or the pressures of business or work, or they may want to confirm that an existing medical problem is the cause rather than their feelings for their partner or their sexuality. Men frequently cited psychological stress, organic disease, and aging as causes for erectile dysfunction in a study of men's sexual beliefs and attitudes[19]. Men's emotional reactions to erectile dysfunction include denial, embarrassment, depression, and acceptance[10]. In keeping with this, men who have intermittent erection problems are less likely to seek treatment[20]. The duration and severity of erectile dysfunction was also determined to be a factor in whether men sought treatment,[11,20,21] suggesting that erectile dysfunction symptoms may not be immediately recognized. When considered together, these factors suggest that for men who answered "no" or "unsure" in the current study and were identified as having erectile dysfunction by IIEF-EF score, the onset of erectile dysfunction symptoms may have been poorly understood, not recognized as erectile dysfunction, or may have been denied.

Men who answered "yes" to the screening question and had erectile dysfunction apparently recognized that they had this condition, but had not been previously diagnosed. This suggests that these men may not have been interested in or were reluctant to seek resolution or treatment for their erectile dysfunction. Some treatment-seeking barriers that may explain why men with recognized erectile dysfunction do not seek treatment for it include the belief that erectile dysfunction is a natural part of aging,[19,20] concern about the side effects of or not wanting to take drugs,[19,20] the belief that nothing can be done about erectile dysfunction,[20] fear that the underlying condition causing erectile dysfunction may be serious,[20] and the cost of treatment[19,20]. One study found that men would prefer to purchase erectile dysfunction medications anonymously or wanted the medication to be available without a prescription,[19] suggesting that men may feel embarrassed about purchasing erectile dysfunction medications.

All men who were screened had comorbidities associated with erectile dysfunction. Pharmaceutical treatments for many of these erectile dysfunction-associated disorders are associated with sexual side effects, including erectile dysfunction. For example, fibrate derivatives used to treat hypercholesterolemia [22] and diuretics and β-blockers for treatment of hypertension have been associated with erectile dysfunction[23]. Drugs for LUTS[24] and depression[25] can also impact sexual health. Men who first experience erectile dysfunction after beginning medication to treat a comorbidity may not consider that they have erectile dysfunction, but may feel that their erectile dysfunction is an adverse effect of their treatment.

## Conclusions

This study found that many men with risk factors associated with erectile dysfunction did have erectile dysfunction, including 54% who had moderate or severe dysfunction; however, these men's awareness of having erectile dysfunction was low. The results suggest that many men may not recognize that they have erectile dysfunction, may possibly deny it, or may not view erectile dysfunction symptoms as a medical problem. Considering the impact that erectile dysfunction has on quality of life and that it may often respond to treatment, erectile dysfunction should be suspected and assessed in men with risk factors, such as cardiovascular disease, diabetes, and LUTS, regardless of their apparent level of awareness of erectile dysfunction.

## Competing interests

Ridwan Shabsigh, MD, has been a consultant or advisor for Pfizer, Eli Lilly, Endo Pharmaceuticals, Boehringer Ingelheim, Bayer Schering Pharma, Dong-A Pharmtech and American Medical Systems and an investigator for Auxilium, Vivus and BioSante. Joel Kaufman, MD, has been a consultant or advisor for Indevus and Eli Lilly; a meeting participant or lecturer for Pfizer Inc and Coloplast; a speakers' bureau member for Novartis and Astellas; and a scientific study/trial investigator for Pfizer, Eli Lilly, Amgen, Solvay, Indevus, and GTX. Michelle Magee, MD, is a consultant for Sanofi-Aventis, a speaker for Sanofi-Aventis, NovoNordisk, Tethys BioScience, and Merck and receives support for investigator-initiated clinical trials from Sanofi-Aventis. She has also received funding for community outreach programs from Johnson & Johnson NovoNordisk, Bayer, Lilly, Pfizer, and Sanofi-Aventis. Dana Creanga was a paid consultant to Pfizer in connection with the development of this manuscript. David Russell and Meeta Budhwani are employees of Pfizer Inc.

The study was funded by Pfizer Inc

## Authors' contributions

All authors made substantial contributions to the acquisition and interpretation of data, critical revision of the manuscript for important intellectual content, and approved the final version for publication. RS made substantial contributions to the conception and design of the study. DLC performed the statistical analysis.

## Pre-publication history

The pre-publication history for this paper can be accessed here:

http://www.biomedcentral.com/1471-2490/10/18/prepub
